# Laparoscopic versus Open Surgery for Colorectal Cancer: A Retrospective Analysis of 163 Patients in a Single Institution

**DOI:** 10.1155/2014/530314

**Published:** 2014-11-23

**Authors:** Abdulkadir Bedirli, Bulent Salman, Osman Yuksel

**Affiliations:** Department of General Surgery, Gazi University Medical Faculty, Besevler, 06510 Ankara, Turkey

## Abstract

*Background*. The present study aimed to compare the clinical outcomes of laparoscopic versus open surgery for colorectal cancers. *Materials and Methods*. The medical records from a total of 163 patients who underwent surgery for colorectal cancers were retrospectively analyzed. Patient's demographic data, operative details and postoperative early outcomes, outpatient follow-up, pathologic results, and stages of the cancer were reviewed from the database. *Results*. The patients who underwent laparoscopic surgery showed significant advantages due to the minimally invasive nature of the surgery compared with those who underwent open surgery, namely, less blood loss, faster postoperative recovery, and shorter postoperative hospital stay (*P* < 0.05). However, laparoscopic surgery for colorectal cancer resulted in a longer operative time compared with open surgery (*P* < 0.05). There were no statistically significant differences between groups for medical complications (*P* > 0.05). Open surgery resulted in more incisional infections and postoperative ileus compared with laparoscopic surgery (*P* < 0.05). There were no differences in the pathologic parameters between two groups (*P* < 0.05). *Conclusions*. These findings indicated that laparoscopic surgery for colorectal cancer had the clear advantages of a minimally invasive surgery and relative disadvantage with longer surgery time and exhibited similar pathologic parameters compared with open surgery.

## 1. Introduction

Colorectal cancer remains the third most common cancer diagnosed and the third most common cause of cancer death in both sexes in industrialized nations [1]. Although many studies have suggested that laparoscopic surgery is superior to open surgery, the acceptance of this technique for colorectal cancer has been rather slow in clinical practice [2, 3]. One of the reasons for the low penetration of this procedure is laparoscopic colon resections which are technically demanding procedures and as such were initially prohibitive for the majority of surgeons [4]. To successfully complete each component of the operation (mobilization of colon, dissection and division of major vessels, removing the specimen, and anastomosis), the surgeon must possess advanced laparoscopic skills, including the ability to operate and recognize anatomy from multiple viewpoints [5].

Concerning the oncologic safety of the laparoscopic approach to colorectal cancers, multiple randomized controlled studies demonstrated that oncological outcomes of laparoscopic surgery were similar to open surgery [6, 7]. The benefits of laparoscopic colorectal surgery are seen in terms of reduced blood loss, less postoperative pain, better pulmonary function, faster return of bowel function, fewer complications, and shorter hospital stay [3, 8]. However, despite the theoretical short-term advantages and equivalent cancer outcomes, adoption rates of laparoscopic colorectal surgery remain low in Europe and USA. The aim of this retrospective review is to assess the feasibility and oncologic adequacy of laparoscopic surgery comparing the operative characteristics and short-term oncological outcomes for laparoscopic surgery with conventional open surgery in patients with colorectal cancer over a period of 3 years in our center.

## 2. Materials and Methods

Between January 2011 and January 2014, we retrospectively analyzed a database containing information about who underwent laparoscopic or open surgery for stage I–III colorectal cancer at Gazi University Hospital, Ankara. Patient's demographic data, operative details and postoperative early outcomes, outpatient follow-up, pathologic results, and stages of the cancer were reviewed from the database. All patients had histologically verified carcinoma of the colon or rectum. The definitive staging in all patients was established via pathological examination of the resected specimens. Operative time was calculated as the time between laparotomy and skin suture for open surgery and pneumoperitoneum induction and port-site closure for laparoscopic surgery.

For this study, we analyzed 65 patients who underwent laparoscopic colorectal surgery (LCRS group) and their results with those of matched 98 patients from our colorectal resection database who had undergone conventional open colorectal surgery (OCRS group) during the same period. Patients with synchronous tumors, tumors located in the transverse colon, stage 0 and IV tumors, and previous malignant tumor and those requiring total colectomy, abdominoperineal resections, or urgent surgery were excluded. All patients and their families were correctly informed and gave their full consent before surgery.

### 2.1. Operation Technique

All operations were performed by the same surgical team that had wide experience with open and laparoscopic colorectal surgery. All patients had bowel preparation with polyethylene glycol, low molecular weight heparin, and intravenous gentamicin plus metronidazole. For laparoscopic resections, pneumoperitoneum with an intra-abdominal pressure between 12 and 14 mmHg was maintained throughout the operation. The first step of the laparoscopic operation is dissection of the colon from medial to lateral and vessel ligation. In right colon operations, specimen is taken out from the incision and the anastomosis is performed extracorporeally with linear stapler. In the left colon and rectum operations, distal resection is performed laparoscopically and proximal end is taken out from the suprapubic incision. After placing the anvil outside, anastomosis is performed intracorporeally. In all patients, a port wound was extended to deliver the specimen under the protection of a plastic ring. A no-touch technique was also used in the open group. Anterior or low anterior resection is performed in rectum tumors according to the localization. Temporary ileostomy is mostly performed in low anterior resection cases. Patients in both groups underwent routine operation according to the complete mesocolic or mesorectal excision principles.

A low-vacuum drainage system was left at the resection site at the end of all operations. Postoperative ileus was defined when insertion of a nasogastric tube was needed and/or there were nausea and vomiting that delayed oral intake for more than 2 days. Patients were discharged when a soft diet was tolerated and they were ambulatory.

### 2.2. Outcome Measures and Statistical Analyses

Clinicopathological characteristics, postoperative outcomes, hospital stay, postoperative morbidity and mortality, and short-term oncological outcomes, including the number of lymph nodes retrieved, the distal margin, radial margin, and pathological staging, were compared. The mean values were compared using paired and unpaired Student's *t*-test. The frequency and distribution were compared using chi-squared test. Statistical significance was assumed when the *P* value was <0.05. These analyses were performed using SPSS 10.0 software (SPSS, Chicago, IL, USA).

## 3. Results

Ninety-eight patients in the OCRS group were compared with 65 patients in the LCRS group. The patient demographic and pathologic characteristics are described in Table 1. Baseline characteristics, including age, sex, surgical risks as assessed by the American Society of Anesthesiologists (ASA), tumor location, and surgical procedures, were similar between the two groups. Protective ileostomy was performed in 23 patients (23%) in OCRS group and 19 patients (29%) in LCRS group. The proportion of patients submitted to neoadjuvant chemoradiotherapy was also similar between the two groups.

The operation time was significantly longer in LCRS group (216 ± 53 min) when compared with OCRS group (172 ± 48 min) (*P* < 0.05). Total amount of blood loss was significantly higher in OCRS group (220 ± 45 mL) when compared with LCRS group (140 ± 35 mL) (*P* < 0.05). There is no conversion to open surgery in LCRS group. Patients in the LCRS group showed a significantly faster postoperative recovery, including faster first flatus time, onset time of the liquid, and normal diet (*P* < 0.05). Despite the similar stay in intensive care unit, total hospital stay was significantly longer for OCRS group than LCRS group (Table 2).

Postoperative details are given in Table 3. No intraoperative complications were reported in both groups. One postoperative death was observed in OCRS group due to a severe pneumosepsis. No significant difference was seen between groups for medical complications (*P* > 0.05). One patient in LCRS group and 9 patients in OCRS group have incisional infections (*P* < 0.05). As for major complications, anastomotic leaks were observed in two patients in LCRS group (one right hemicolectomy and one low anterior resection) and three patients in OCRS (one right hemicolectomy and two low anterior resections) (*P* > 0.05). Two patients in LCRS group and 5 patients in OCRS group suffered postoperative ileus (*P* < 0.05).

All the resections in both groups were performed to remove a malignancy. Most frequent histologic types were moderately differentiated adenocarcinoma in both groups. The mean number of lymph nodes harvested was comparable between LCRS and OCRS groups, 19 ± 7 versus 23 ± 8, respectively. The ratio of patients with stage III tumors was relatively higher in the OCRS group. However, none of these pathologic parameters showed statistical differences between two groups (*P* > 0.05) (Table 4).

## 4. Discussion

This study compares the short-term surgical outcomes of 163 consecutive patients undergoing open or laparoscopic surgery for colorectal cancer. Compared with open surgery, laparoscopic surgery at our institution was associated with slightly longer operative time, significantly faster postoperative recovery, lower incisional infections and postoperative ileus, and similar pathologic results.

Laparoscopic colorectal surgery in particular has raised the last decade after multiple, large, randomized, controlled trials in colorectal cancer have displayed that this approach is safe and with equal oncological results as open surgery [6, 9, 10]. Despite similar cancer outcomes and postoperative advantages in laparoscopic surgery, most colorectal cancers are treated by open surgery. The main barrier to widespread adoption has been the technical difficulty of these operations [4]. Laparoscopic colorectal surgery demands not only the experiences in open surgery of colon and rectum but also skills in advanced laparoscopic techniques. At the beginning, operation time is the one of the much discussed subjects in laparoscopic surgery. When 4125 cases which were collected from the related randomised clinical studies were evaluated, it was seen that the operation time in laparoscopic surgery is significantly longer than open surgery [11]. When we look at the progress of the laparoscopic surgery teams, it is clearly seen that the operation time is significantly decreased with the experience [12]. In our study, the mean time difference between laparoscopy and open surgery was around 40 minutes. In previous studies, it was found that intraoperatively the amount of blood loss in laparoscopic surgery was significantly less than in the open surgery [2, 11]. Although measurement of intraoperative blood loss is hard to standardize, it is obvious that blood loss is minimal because of high definition and large view and fine dissection in laparoscopic surgery. Similar to the previous studies, the amount of blood loss in OCRS group was significantly higher than the LCRS group in our study.

As being a difficult operation, conversion to open surgery can be an option during laparoscopic colorectal surgery in some instances. The rate of conversion to open surgery has been reported between 10 and 15% in different series [13, 14]. Restrictive factors for the reasons to conversion to open surgery are obesity, type of surgery, ASA scores of the patients, large tumor, intra-abdominal adhesions, technical problems, organ injuries, being unable to see the operation area, being unable to free the structures, unsafe tumor resection site, and difficulties in anastomosis. There has not been any conversion to open surgery in our study. Surgical experience and careful patient selection can be accepted as the reasons for the lack of conversion to open surgery. In our study, anastomotic leak rate was low overall (3%), with two patients in the LCRS group and three patients in the OCRS group. Leak rates for open surgery were from 2.4% to 6.8% [15, 16]. In meta-analyses comparing outcomes in laparoscopic colorectal surgery by Kelly and colleagues, the overall rate of anastomotic leak rate was 2.7% [17]. It is well documented that postoperative complications are decreasing with the increased surgical experience especially anastomosis leakage, intra-abdominal infection, and mortality [4, 5].

Large number of randomized controlled trials comparing laparoscopic to open surgery for colon cancer have established better short-term results, less pain, shorter length of stay, faster return of bowel function, and equivalent oncological outcomes [2, 3]. Laparoscopic rectal surgery is still developing with promising short-term benefit, although depending on the skills and techniques of the surgeon [5]. According to the COLOR study, the increased number of the patients treated with laparoscopy at an institution closely related with the improved short-term results of the operations [8]. In our study, the benefits of laparoscopic colorectal surgery are seen in terms of reduced blood loss, faster return of bowel function, fewer surgical complications, and shorter hospital stay.

After the initial description in 1991, several reports of laparoscopic colectomy for colorectal cancer were described. Significant concerns regarding this approach surfaced when minimally invasive techniques applied to colorectal malignancy lead to increased surgical complications and worse cancer outcomes compared to conventional open approaches. Although well-defined method of laparoscopic surgery for colorectal cancer, surgery should be performed by expert surgeons in selected patients. One of the important parameters in oncological surgery is dissected lymph nodes. At least 12 lymph nodes should be resected for a sufficient lymph node dissection. The status of lymph nodes is closely related with prognosis and the adjuvant treatment protocol. For this reason, the number of resected lymph nodes is an important oncological parameter in laparoscopy also. In our study, mean 19 and 23 lymph nodes were resected from the patients in the LCRS and OCRS groups, respectively. Sufficient number of resected lymph nodes shows appropriate mesorectum and mesocolic dissection in our study. In several previous studies, the number of resected lymph nodes is found to be increased with the increased experience [18, 19]. Similarly in our study the number of resected lymph nodes significantly increased after the learning curve period. In the COST and COLOR studies, it is advised to operate the patients with small tumors (T1, T2) or easy cases like sigmoid tumors in learning curve periods and then operate big tumors (T3, T4) and difficult cases like low anterior resections when more experience has been gained [20, 21].

## 5. Conclusion

It has been demonstrated in the literature that laparoscopic colorectal surgery is safe and feasible, with an oncological adequacy comparable to the open approach. But apart from these published data, open surgery is still performed more frequently worldwide. So we believe that it is important to share clinics own experiences on laparoscopic colorectal surgery. Supporting the literature results of this study showed that laparoscopic colorectal surgery is convenient and less invasive and probably could be the first choice of intervention for colorectal cancers. In our series, the operating time represents a disadvantage for laparoscopic surgery; however, we think that this might be overcome with increased experience.

## Figures and Tables

**Table 1:** Patient and tumor characteristics.

	LCRS group (*n* = 65)	OCRS group (*n* = 98)	*P* ^*^
Age (y), mean ± SD	57.7 ± 9.2	62.3 ± 11.1	NS
Gender (male/female)	38/27	56/42	NS
ASA (%)			NS
I	16 (25)	22 (22)	
II	29 (44)	45 (46)	
III	20 (31)	31 (32)	
Tumor distribution (%)			NS
Right colon	17 (26)	31 (32)	
Left colon	6 (9)	7 (7)	
Sigmoid colon	13 (20)	24 (24)	
Rectum	29 (45)	36 (37)	
Operation (%)			NS
Right hemicolectomy	17 (26)	31 (32)	
Left hemicolectomy	6 (9)	7 (7)	
Sigmoid resection	11 (17)	10 (10)	
Anterior resection	9 (14)	21 (21)	
Low anterior resection	22 (34)	29 (30)	
Protective ileostomy: yes (%)	19 (29)	23 (23)	NS
Neoadjuvant chemoradiotherapy: yes (%)	17 (26)	22 (22)	NS

NS: not significant. ASA: American Society of Anesthesiologist.

^*^Chi-square test.

**Table 2:** Operative and postoperative results of the two patient groups.

	LCRS group (*n* = 65)	OCRS group (*n* = 98)	*P*
Operation time (min), mean ± SD	216 ± 53	172 ± 48	0.039^†^
Operative blood loss (mL), mean ± SD	140 ± 35	220 ± 45	0.040^†^
Time to flatus (d), mean ± SD	1.6 ± 1.4	2.3 ± 1.7	0.014^†^
Time to liquid diet (d), mean ± SD	2.3 ± 1.9	2.9 ± 2.3	0.032^†^
Time to normal diet (d), mean ± SD	3.4 ± 2.2	4.2 ± 2.8	0.030^†^
Stay in ICU (d), mean ± SD	2.3 ± 2.7	2.4 ± 4.4	NS
Total hospital stay (d), mean ± SD	4.5 ± 4.0	6.2 ± 5.3	0.028^†^

NS: not significant. ICU: intensive care unit.

^†^Student's *t*-test.

**Table 3:** Postoperative morbidity and mortality.

	LCRS group (*n* = 65)	OCRS group (*n* = 98)	*P*
Incision infection	1 (2)	9 (9)	0.021^†^
Anastomotic leakage	2 (3)	3 (3)	NS
Postoperative ileus	2 (3)	5 (5)	0.021^†^
Major medical complication (%)			NS
Pneumonia	3 (5)	3 (3)	
Cardiac decompensation	1 (2)	—	
Myocardial infarction	1 (2)	1 (1)	
Renal failure	—	1 (1)	
Cerebrovascular accident	—	1 (1)	
Death in hospital	—	1 (1)	NS

NS: not significant.

^†^Student's *t*-test.

**Table 4:** Comparison of the pathological parameters in two groups.

	LCRS group (*n* = 65)	OCRS group (*n* = 98)	*P*
Number of lymph nodes, mean ± SD	19 ± 7	23 ± 8	NS
Histology (%)			NS
Well-differentiated adenocarcinoma	16 (25)	20 (20)	
Moderately differentiated adenocarcinoma	29 (45)	41 (42)	
Poorly differentiated adenocarcinoma	17 (26)	31 (32)	
Others	3 (4)	6 (6)	
Tumor stage (TNM) (%)			NS
I	7 (11)	7 (7)	
II-A	14 (21)	17 (17)	
II-B	17 (26)	27 (28)	
III-A	9 (14)	19 (20)	
III-B	11 (17)	16 (16)	
III-C	7 (11)	12 (12)	

NS: not significant.
